# Supplementary material for: Mapping the evolution of fertility support policies in China: A content and instrumental analysis
Source: PLoS One. 2025 Oct 9;20(10):e0332137. doi: 10.1371/journal.pone.0332137 (PMC12510515; doi:10.1371/journal.pone.0332137)
Supplement: S1 Appendix — (ZIP) [file pone.0332137.s001.zip › S1 Appendix. 226 original policy documents/150-中华人民共和国妇女权益保障法(2018修正)(FBM-CLI-1-324963).doc]

中华人民共和国妇女权益保障法(2018修正)

**发布部门：** [全国人大常委会](https://www.pkulaw.com/law/chl?Aggs.IssueDepartment=201)

**发文字号：**中华人民共和国主席令第16号

**发布日期：**2018.10.26

**实施日期：**2018.10.26

**时效性：** [现行有效](https://www.pkulaw.com/law/chl?Aggs.TimelinessDic=01)

**效力级别：** [法律](https://www.pkulaw.com/law/chl?Aggs.EffectivenessDic=XA0101)

**法规类别：** [老少妇幼残保护](https://www.pkulaw.com/law/chl?Aggs.Category=01604)

[2005-2018编注](https://www.pkulaw.com/history/compare/编注-122.html) [1992-2005编注](https://www.pkulaw.com/history/compare/编注-121.html)

中华人民共和国妇女权益保障法
（1992年4月3日第七届全国人民代表大会第五次会议通过　根据2005年8月28日第十届全国人民代表大会常务委员会第十七次会议《[关于修改〈中华人民共和国妇女权益保障法〉的决定](https://www.pkulaw.com/chl/3feb220e540f2e2dbdfb.html#class=alink)》第一次修正　根据2018年10月26日第十三届全国人民代表大会常务委员会第六次会议《[关于修改〈中华人民共和国野生动物保护法〉等十五部法律的决定](https://www.pkulaw.com/chl/4c2a4217ea825041bdfb.html#class=alink)》第二次修正）

目　　录

　　第一章　总　　则
　　第二章　政治权利
　　第三章　文化教育权益
　　第四章　劳动和社会保障权益
　　第五章　财产权益
　　第六章　人身权利
　　第七章　婚姻家庭权益
　　第八章　法律责任
　　第九章　附　　则

第一章　总　　则

　　**第一条**　为了保障妇女的合法权益，促进男女平等，充分发挥妇女在社会主义现代化建设中的作用，根据[宪法](https://www.pkulaw.com/chl/7c7e81f43957c58bbdfb.html#class=alink)和我国的实际情况，制定本法。

　　**第二条**　妇女在政治的、经济的、文化的、社会的和家庭的生活等各方面享有同男子平等的权利。

　　实行男女平等是国家的基本国策。国家采取必要措施，逐步完善保障妇女权益的各项制度，消除对妇女一切形式的歧视。

　　国家保护妇女依法享有的特殊权益。

　　禁止歧视、虐待、遗弃、残害妇女。

　　**第三条**　国务院制定[中国妇女发展纲要](https://www.pkulaw.com/chl/0fe9880c331fcc87bdfb.html#class=alink)，并将其纳入国民经济和社会发展规划。

　　县级以上地方各级人民政府根据[中国妇女发展纲要](https://www.pkulaw.com/chl/0fe9880c331fcc87bdfb.html#class=alink)，制定本行政区域的妇女发展规划，并将其纳入国民经济和社会发展计划。

　　**第四条**　保障妇女的合法权益是全社会的共同责任。国家机关、社会团体、企业事业单位、城乡基层群众性自治组织，应当依照本法和有关法律的规定，保障妇女的权益。

　　国家采取有效措施，为妇女依法行使权利提供必要的条件。

　　**第五条**　国家鼓励妇女自尊、自信、自立、自强，运用法律维护自身合法权益。

　　妇女应当遵守国家法律，尊重社会公德，履行法律所规定的义务。

　　**第六条**　各级人民政府应当重视和加强妇女权益的保障工作。

　　县级以上人民政府负责妇女儿童工作的机构，负责组织、协调、指导、督促有关部门做好妇女权益的保障工作。

　　县级以上人民政府有关部门在各自的职责范围内做好妇女权益的保障工作。

　　**第七条**　中华全国妇女联合会和地方各级妇女联合会依照法律和[中华全国妇女联合会章程](https://www.pkulaw.com/chl/c75ed501dd22fce0bdfb.html#class=alink)，代表和维护各族各界妇女的利益，做好维护妇女权益的工作。

　　工会、共产主义青年团，应当在各自的工作范围内，做好维护妇女权益的工作。

　　**第八条**　对保障妇女合法权益成绩显著的组织和个人，各级人民政府和有关部门给予表彰和奖励。

第二章　政治权利

　　**第九条**　国家保障妇女享有与男子平等的政治权利。

　　**第十条**　妇女有权通过各种途径和形式，管理国家事务，管理经济和文化事业，管理社会事务。

　　制定法律、法规、规章和公共政策，对涉及妇女权益的重大问题，应当听取妇女联合会的意见。

　　妇女和妇女组织有权向各级国家机关提出妇女权益保障方面的意见和建议。

　　**第十一条**　妇女享有与男子平等的选举权和被选举权。

　　全国人民代表大会和地方各级人民代表大会的代表中，应当有适当数量的妇女代表。国家采取措施，逐步提高全国人民代表大会和地方各级人民代表大会的妇女代表的比例。

　　居民委员会、村民委员会成员中，妇女应当有适当的名额。

　　**第十二条**　国家积极培养和选拔女干部。

　　国家机关、社会团体、企业事业单位培养、选拔和任用干部，必须坚持男女平等的原则，并有适当数量的妇女担任领导成员。

　　国家重视培养和选拔少数民族女干部。

　　**第十三条**　中华全国妇女联合会和地方各级妇女联合会代表妇女积极参与国家和社会事务的民主决策、民主管理和民主监督。

　　各级妇女联合会及其团体会员，可以向国家机关、社会团体、企业事业单位推荐女干部。

　　**第十四条**　对于有关保障妇女权益的批评或者合理建议，有关部门应当听取和采纳；对于有关侵害妇女权益的申诉、控告和检举，有关部门必须查清事实，负责处理，任何组织或者个人不得压制或者打击报复。

第三章　文化教育权益

　　**第十五条**　国家保障妇女享有与男子平等的文化教育权利。

　　**第十六条**　学校和有关部门应当执行国家有关规定，保障妇女在入学、升学、毕业分配、授予学位、派出留学等方面享有与男子平等的权利。

　　学校在录取学生时，除特殊专业外，不得以性别为由拒绝录取女性或者提高对女性的录取标准。

　　**第十七条**　学校应当根据女性青少年的特点，在教育、管理、设施等方面采取措施，保障女性青少年身心健康发展。

　　**第十八条**　父母或者其他监护人必须履行保障适龄女性儿童少年接受义务教育的义务。

　　除因疾病或者其他特殊情况经当地人民政府批准的以外，对不送适龄女性儿童少年入学的父母或者其他监护人，由当地人民政府予以批评教育，并采取有效措施，责令送适龄女性儿童少年入学。

　　政府、社会、学校应当采取有效措施，解决适龄女性儿童少年就学存在的实际困难，并创造条件，保证贫困、残疾和流动人口中的适龄女性儿童少年完成义务教育。

　　**第十九条**　各级人民政府应当依照规定把扫除妇女中的文盲、半文盲工作，纳入扫盲和扫盲后继续教育规划，采取符合妇女特点的组织形式和工作方法，组织、监督有关部门具体实施。

　　**第二十条**　各级人民政府和有关部门应当采取措施，根据城镇和农村妇女的需要，组织妇女接受职业教育和实用技术培训。

　　**第二十一条**　国家机关、社会团体和企业事业单位应当执行国家有关规定，保障妇女从事科学、技术、文学、艺术和其他文化活动，享有与男子平等的权利。

第四章　劳动和社会保障权益

　　**第二十二条**　国家保障妇女享有与男子平等的劳动权利和社会保障权利。

　　**第二十三条**　各单位在录用职工时，除不适合妇女的工种或者岗位外，不得以性别为由拒绝录用妇女或者提高对妇女的录用标准。

　　各单位在录用女职工时，应当依法与其签订劳动（聘用）合同或者服务协议，劳动（聘用）合同或者服务协议中不得规定限制女职工结婚、生育的内容。

　　禁止录用未满十六周岁的女性未成年人，国家另有规定的除外。

　　**第二十四条**　实行男女同工同酬。妇女在享受福利待遇方面享有与男子平等的权利。

　　**第二十五条**　在晋职、晋级、评定专业技术职务等方面，应当坚持男女平等的原则，不得歧视妇女。

　　**第二十六条**　任何单位均应根据妇女的特点，依法保护妇女在工作和劳动时的安全和健康，不得安排不适合妇女从事的工作和劳动。

　　妇女在经期、孕期、产期、哺乳期受特殊保护。

　　**第二十七条**　任何单位不得因结婚、怀孕、产假、哺乳等情形，降低女职工的工资，辞退女职工，单方解除劳动（聘用）合同或者服务协议。但是，女职工要求终止劳动（聘用）合同或者服务协议的除外。

　　各单位在执行国家退休制度时，不得以性别为由歧视妇女。

　　**第二十八条**　国家发展社会保险、社会救助、社会福利和医疗卫生事业，保障妇女享有社会保险、社会救助、社会福利和卫生保健等权益。

　　国家提倡和鼓励为帮助妇女开展的社会公益活动。

　　**第二十九条**　国家推行生育保险制度，建立健全与生育相关的其他保障制度。

　　地方各级人民政府和有关部门应当按照有关规定为贫困妇女提供必要的生育救助。

第五章　财产权益

　　**第三十条**　国家保障妇女享有与男子平等的财产权利。

　　**第三十一条**　在婚姻、家庭共有财产关系中，不得侵害妇女依法享有的权益。

　　**第三十二条**　妇女在农村土地承包经营、集体经济组织收益分配、土地征收或者征用补偿费使用以及宅基地使用等方面，享有与男子平等的权利。

　　**第三十三条**　任何组织和个人不得以妇女未婚、结婚、离婚、丧偶等为由，侵害妇女在农村集体经济组织中的各项权益。

　　因结婚男方到女方住所落户的，男方和子女享有与所在地农村集体经济组织成员平等的权益。

　　**第三十四条**　妇女享有的与男子平等的财产继承权受法律保护。在同一顺序法定继承人中，不得歧视妇女。

　　丧偶妇女有权处分继承的财产，任何人不得干涉。

　　**第三十五条**　丧偶妇女对公、婆尽了主要赡养义务的，作为公、婆的第一顺序法定继承人，其继承权不受子女代位继承的影响。

第六章　人身权利

　　**第三十六条**　国家保障妇女享有与男子平等的人身权利。

　　**第三十七条**　妇女的人身自由不受侵犯。禁止非法拘禁和以其他非法手段剥夺或者限制妇女的人身自由；禁止非法搜查妇女的身体。

　　**第三十八条**　妇女的生命健康权不受侵犯。禁止溺、弃、残害女婴；禁止歧视、虐待生育女婴的妇女和不育的妇女；禁止用迷信、暴力等手段残害妇女；禁止虐待、遗弃病、残妇女和老年妇女。

　　**第三十九条**　禁止拐卖、绑架妇女；禁止收买被拐卖、绑架的妇女；禁止阻碍解救被拐卖、绑架的妇女。

　　各级人民政府和公安、民政、劳动和社会保障、卫生等部门按照其职责及时采取措施解救被拐卖、绑架的妇女，做好善后工作，妇女联合会协助和配合做好有关工作。任何人不得歧视被拐卖、绑架的妇女。

　　**第四十条**　禁止对妇女实施性骚扰。受害妇女有权向单位和有关机关投诉。

　　**第四十一条**　禁止卖淫、嫖娼。

　　禁止组织、强迫、引诱、容留、介绍妇女卖淫或者对妇女进行猥亵活动。

　　禁止组织、强迫、引诱妇女进行淫秽表演活动。

　　**第四十二条**　妇女的名誉权、荣誉权、隐私权、肖像权等人格权受法律保护。

　　禁止用侮辱、诽谤等方式损害妇女的人格尊严。禁止通过大众传播媒介或者其他方式贬低损害妇女人格。未经本人同意，不得以营利为目的，通过广告、商标、展览橱窗、报纸、期刊、图书、音像制品、电子出版物、网络等形式使用妇女肖像。

第七章　婚姻家庭权益

　　**第四十三条**　国家保障妇女享有与男子平等的婚姻家庭权利。

　　**第四十四条**　国家保护妇女的婚姻自主权。禁止干涉妇女的结婚、离婚自由。

　　**第四十五条**　女方在怀孕期间、分娩后一年内或者终止妊娠后六个月内，男方不得提出离婚。女方提出离婚的，或者人民法院认为确有必要受理男方离婚请求的，不在此限。

　　**第四十六条**　禁止对妇女实施家庭暴力。

　　国家采取措施，预防和制止家庭暴力。

　　公安、民政、司法行政等部门以及城乡基层群众性自治组织、社会团体，应当在各自的职责范围内预防和制止家庭暴力，依法为受害妇女提供救助。

　　**第四十七条**　妇女对依照法律规定的夫妻共同财产享有与其配偶平等的占有、使用、收益和处分的权利，不受双方收入状况的影响。

　　夫妻书面约定婚姻关系存续期间所得的财产归各自所有，女方因抚育子女、照料老人、协助男方工作等承担较多义务的，有权在离婚时要求男方予以补偿。

　　**第四十八条**　夫妻共有的房屋，离婚时，分割住房由双方协议解决；协议不成的，由人民法院根据双方的具体情况，按照照顾子女和女方权益的原则判决。夫妻双方另有约定的除外。

　　夫妻共同租用的房屋，离婚时，女方的住房应当按照照顾子女和女方权益的原则解决。

　　**第四十九条**　父母双方对未成年子女享有平等的监护权。

　　父亲死亡、丧失行为能力或者有其他情形不能担任未成年子女的监护人的，母亲的监护权任何人不得干涉。

　　**第五十条**　离婚时，女方因实施绝育手术或者其他原因丧失生育能力的，处理子女抚养问题，应在有利子女权益的条件下，照顾女方的合理要求。

　　**第五十一条**　妇女有按照国家有关规定生育子女的权利，也有不生育的自由。

　　育龄夫妻双方按照国家有关规定计划生育，有关部门应当提供安全、有效的避孕药具和技术，保障实施节育手术的妇女的健康和安全。

　　国家实行婚前保健、孕产期保健制度，发展母婴保健事业。各级人民政府应当采取措施，保障妇女享有计划生育技术服务，提高妇女的生殖健康水平。

第八章　法律责任

　　**第五十二条**　妇女的合法权益受到侵害的，有权要求有关部门依法处理，或者依法向仲裁机构申请仲裁，或者向人民法院起诉。

　　对有经济困难需要法律援助或者司法救助的妇女，当地法律援助机构或者人民法院应当给予帮助，依法为其提供法律援助或者司法救助。

　　**第五十三条**　妇女的合法权益受到侵害的，可以向妇女组织投诉，妇女组织应当维护被侵害妇女的合法权益，有权要求并协助有关部门或者单位查处。有关部门或者单位应当依法查处，并予以答复。

　　**第五十四条**　妇女组织对于受害妇女进行诉讼需要帮助的，应当给予支持。

　　妇女联合会或者相关妇女组织对侵害特定妇女群体利益的行为，可以通过大众传播媒介揭露、批评，并有权要求有关部门依法查处。

　　**第五十五条**　违反本法规定，以妇女未婚、结婚、离婚、丧偶等为由，侵害妇女在农村集体经济组织中的各项权益的，或者因结婚男方到女方住所落户，侵害男方和子女享有与所在地农村集体经济组织成员平等权益的，由乡镇人民政府依法调解；受害人也可以依法向农村土地承包仲裁机构申请仲裁，或者向人民法院起诉，人民法院应当依法受理。

　　**第五十六条**　违反本法规定，侵害妇女的合法权益，其他法律、法规规定行政处罚的，从其规定；造成财产损失或者其他损害的，依法承担民事责任；构成犯罪的，依法追究刑事责任。

　　**第五十七条**　违反本法规定，对侵害妇女权益的申诉、控告、检举，推诿、拖延、压制不予查处，或者对提出申诉、控告、检举的人进行打击报复的，由其所在单位、主管部门或者上级机关责令改正，并依法对直接负责的主管人员和其他直接责任人员给予行政处分。

　　国家机关及其工作人员未依法履行职责，对侵害妇女权益的行为未及时制止或者未给予受害妇女必要帮助，造成严重后果的，由其所在单位或者上级机关依法对直接负责的主管人员和其他直接责任人员给予行政处分。

　　违反本法规定，侵害妇女文化教育权益、劳动和社会保障权益、人身和财产权益以及婚姻家庭权益的，由其所在单位、主管部门或者上级机关责令改正，直接负责的主管人员和其他直接责任人员属于国家工作人员的，由其所在单位或者上级机关依法给予行政处分。

　　**第五十八条**　违反本法规定，对妇女实施性骚扰或者家庭暴力，构成违反治安管理行为的，受害人可以提请公安机关对违法行为人依法给予行政处罚，也可以依法向人民法院提起民事诉讼。

　　**第五十九条**　违反本法规定，通过大众传播媒介或者其他方式贬低损害妇女人格的，由文化、广播电视、电影、新闻出版或者其他有关部门依据各自的职权责令改正，并依法给予行政处罚。

第九章　附　　则

　　**第六十条**　省、自治区、直辖市人民代表大会常务委员会可以根据本法制定实施办法。

　　民族自治地方的人民代表大会，可以依据本法规定的原则，结合当地民族妇女的具体情况，制定变通的或者补充的规定。自治区的规定，报全国人民代表大会常务委员会批准后生效；自治州、自治县的规定，报省、自治区、直辖市人民代表大会常务委员会批准后生效，并报全国人民代表大会常务委员会备案。

　　**第六十一条**　本法自1992年10月1日起施行。

© 北大法宝：（[www.pkulaw.com](https://www.pkulaw.com/)) 专业提供法律信息、法学知识和法律软件领域各类解决方案。北大法宝为您提供丰富的参考资料，正式引用法规条文时请与标准文本核对。
欢迎查看所有[产品和服务](https://www.pkulaw.com/helps/2.html)。法宝快讯：[如何快速找到您需要的检索结果？](https://www.pkulaw.com/helps/70.html)[法宝](https://www.pkulaw.com/helps/69.html)V6有何新特色？

扫描二维码阅读原文

**原文链接：**<https://www.pkulaw.com/chl/f20e7cd055022d39bdfb.html>
